# Machine Learning Identifies Cellular and Exosomal MicroRNA Signatures of Lyssavirus Infection in Human Stem Cell-Derived Neurons

**DOI:** 10.3389/fcimb.2021.783140

**Published:** 2021-12-24

**Authors:** Ryan J. Farr, Nathan Godde, Christopher Cowled, Vinod Sundaramoorthy, Diane Green, Cameron Stewart, John Bingham, Carmel M. O’Brien, Megan Dearnley

**Affiliations:** ^1^ Commonwealth Scientific and Industrial Research Organisation (CSIRO) Australian Animal Health Laboratory at the Australian Centre for Disease Preparedness, Geelong, VIC, Australia; ^2^ Commonwealth Scientific and Industrial Research Organisation (CSIRO) Health and Biosecurity at the Australian Centre for Disease Preparedness, Geelong, VIC, Australia; ^3^ Commonwealth Scientific and Industrial Research Organisation (CSIRO) Manufacturing, Clayton, VIC, Australia; ^4^ Australian Regenerative Medicine Institute, Monash University, Clayton, VIC, Australia

**Keywords:** microRNA, lyssavirus, stem cells, neural, neurons, biomarker, machine learning

## Abstract

Despite being vaccine preventable, rabies (lyssavirus) still has a significant impact on global mortality, disproportionally affecting children under 15 years of age. This neurotropic virus is deft at avoiding the immune system while travelling through neurons to the brain. Until recently, research efforts into the role of non-coding RNAs in rabies pathogenicity and detection have been hampered by a lack of human *in vitro* neuronal models. Here, we utilized our previously described human stem cell-derived neural model to investigate the effect of lyssavirus infection on microRNA (miRNA) expression in human neural cells and their secreted exosomes. Conventional differential expression analysis identified 25 cellular and 16 exosomal miRNAs that were significantly altered (FDR adjusted P-value <0.05) in response to different lyssavirus strains. Supervised machine learning algorithms determined 6 cellular miRNAs (miR-99b-5p, miR-346, miR-5701, miR-138-2-3p, miR-651-5p, and miR-7977) were indicative of lyssavirus infection (100% accuracy), with the first four miRNAs having previously established roles in neuronal function, or panic and impulsivity-related behaviors. Another 4-miRNA signatures in exosomes (miR-25-3p, miR-26b-5p, miR-218-5p, miR-598-3p) can independently predict lyssavirus infected cells with >99% accuracy. Identification of these robust lyssavirus miRNA signatures offers further insight into neural lineage responses to infection and provides a foundation for utilizing exosome miRNAs in the development of next-generation molecular diagnostics for rabies.

## Introduction

Rabies virus (Family Rhabdoviridae, genus Lyssavirus) is among the most lethal viruses that invade and infect the central nervous system (CNS), leading to an estimated 59,000 deaths annually worldwide (Knobel et al., 2005; Schnell et al., 2010; Hampson et al., 2015; Olugasa et al., 2020; Abdulmoghni et al., 2021). As rabies is a neglected tropical disease that predominantly impacts economically poor and non-industrialized countries, the actual burden of this disease is likely to be significantly under-reported (Taylor et al., 2017; WHO, 2018). Classical rabies virus is the most commonly known member of Lyssavirus genus (Troupin et al., 2016), however there are 15 other genetically related viruses within the genus, most of which have been isolated from bats (Delmas et al., 2008; Rupprecht et al., 2017; Shipley et al., 2019).

Transmission of the virus occurs following intramuscular exposure, usually through an animal bite. From the wound site, the virus migrates to the brain *via* the peripheral nervous system in an asymptomatic pre-clinical phase, typically taking between two weeks and three months to occur (Schnell et al., 2010). Once resident in the CNS, the virus causes encephalomyelitis and manifests in neurological symptoms including, seizures, confusion, aggression, muscle weakness, paralysis, and ultimately culminates in death. Treatment of patients presenting with clinical signs is limited to palliative care, or in rare cases, implementation of the “Milwaukee protocol” is used to induce a therapeutic coma and an anti-excitotoxic strategy while the native immune response matures (Willoughby et al., 2005; Ledesma et al., 2020). However, more contemporary review of these methods has cast doubt on scientifically proven validity of this approach (Wilde and Hemachudha, 2015; Zeiler and Jackson, 2016).

Administration of a rabies vaccine and/or post-exposure prophylaxis treatment (PEP) has proven efficacy in combatting the infection in the pre-clinical phase (Sreenivasan et al., 2019). Whilst this is easily accessed throughout wealthy countries, in developing countries access to vaccination and PEP is limited and often very expensive. Many people in these resource-poor situations are left to lives with the progression and outcome of the disease.

An absence of diagnostic tests for pre-clinical rabies infection further compounds the difficulty treating and managing the risk of clinical disease onset. The virus can efficiently evade antigen presentation to the immune system during replication and transit between neurons (Ito et al., 2016), so traditional serological and molecular diagnostics are often unable to detect pre-clinical viral infection and sensitivity to serological tests is as low as 20% in unvaccinated patients (Mahadevan et al., 2016). The current gold standard diagnostic test for rabies utilizes post-mortem brain smears to fluorescently detect viral antigens, and is, for obvious reasons, of little help outside disease surveillance and reporting. Additionally, other etiologies (both infectious and non-infectious) may result in a similar clinical presentation, which can result in misdiagnosis, especially in regions where human rabies is less common (Fooks et al., 2017). Therefore, the development of diagnostic or testing protocols for detection of rabies virus infection needs to circumvent the pitfalls of “traditional” approaches and instead consider a more novel approach to understanding and detecting this evasive virus.

MicroRNAs (miRNAs) are central players in cellular development and function. They have established roles in brain development and neuronal communication and are important in the development of neurodegenerative disorders (Thomas et al., 2018; Cho et al., 2019; Garcia-Fonseca et al., 2021). These small (20-22nt) non-coding RNAs regulate endogenous gene expression and display rapid changes in response to viral infection (within 3 hours *in vitro* and 24 hours *in vivo* when challenged with Hendra virus) (Stewart et al., 2013). By examining the changes in miRNA expression during viral infection, we can gain insights into the molecular pathways of viral pathogenesis and further explore potential pro- and anti-viral targets (Foo et al., 2016; Trobaugh and Klimstra, 2017).

Recent studies have highlighted miRNAs as potential biomarkers for a wide range of infectious diseases, including viral infection (Cowled et al., 2017; Biswas et al., 2019; Tribolet et al., 2020). MicroRNAs are present in all reported biofluids, including plasma/serum, urine, milk, and saliva, which allows minimally invasive sampling for the detection of localized disease. MicroRNAs are also resistant to degradation by endonucleases, temperature, and pH, thus providing a robust and promising biomarker for diagnostic applications (Chen et al., 2008; Tribolet et al., 2020).

Furthermore, miRNAs are often packaged into exosomes (extracellular vesicles 30-100 nm in size) and their abundance changes in a wide range of disease states, including neurodegenerative and infectious diseases (Bellingham et al., 2012; Baluni et al., 2018; Lyu et al., 2019; Wang et al., 2019; Hornung et al., 2020). As exosomes are also found in a range of biological fluids (including blood, urine, saliva, cerebrospinal fluid and synovial fluid), there is a growing interest in exploring exosomes and their contents as non-invasive biomarkers for the early detection and prognosis of diseases (Zhang and Yang, 2018). Interestingly, an increase in exosome production has been reported to correlate with rabies infection (Wang et al., 2019), however an examination of their miRNA cargo has not been performed.

MicroRNA expression profiling has been documented in mouse brain tissue following infection with rabies virus (Zhao et al., 2012a; Zhao et al., 2012b; Shi et al., 2014), although analysis of lyssavirus-induced miRNA changes in human neurons has not been explored due to limitations in experimental platforms available. Previously, we have generated and characterized human stem cell-derived neural models for the investigation of rabies pathogenesis (Sundaramoorthy et al., 2020a). This approach allows ethical, reproducible, high-throughput, and relevant investigations of lyssavirus infections directly on a human cell system.

In this study we have utilized one of these models to investigate the lyssavirus-induced changes in neural miRNAs, providing insights into the molecular mechanisms of pathogenesis of this deadly neurotropic virus. Using this model, we have also examined virus-mediated miRNAs released in exosomes as a putative source of disease biomarkers for future diagnostic applications. Implementation of bioinformatics and advanced machine learning (ML) techniques in this study have aided the discovery, refinement, and validation of these host biomarkers, and allowed a direct comparison to the conventional differential expression (DE) analysis of miRNA signatures. Together, these novel cell culture and computational platforms have provided a new way of assessing the host-virus response of lyssavirus infection in human neurons and provides a modern appraisal on the validity of miRNA signatures as biomarkers of lyssavirus infection.

## Methods

### Ethics Statement

Human ethics: All work using human pluripotent-derived neural stem and progenitor cells (hNPCs) was carried out in accordance with Australia’s National Health and Medical Research Council (NHMRC) ‘National Statement on Ethical Conduct in Human Research’ (2007, updated 2018), the ‘Australian Code for the Responsible Conduct of Research’ (2007, updated 2018), and with approval from the Commonwealth Scientific and Industrial Research Organization (CSIRO) Health and Medical Research Ethics Committee (LR 16/2017 29/11/2017).

### Cell Culture and Lyssavirus Infection

HDF51i-509 neural progenitor cells (NPCs) were maintained in culture and terminally differentiated as previously described for at least 25 days (Sundaramoorthy et al., 2020a). Lyssavirus culture and infections were also performed as previously described (Sundaramoorthy et al., 2020a). To investigate the host molecular response to lyssavirus infection we utilized several different strains, including a laboratory-adapted CVS-11 strain, an Australian bat lyssavirus strain isolated from an infected horse (H.ABLV), and two rabies viruses, one from a Canadian silver-haired bat (SHBRV), and the other from a Zimbabwean dog (Z.Dog). Briefly, infections were conducted using human stem cell-derived neural cultures differentiated in a 24-well plate for 25-32 days with an MOI of 1 (based on the viral titre determined in BHK-1 cells). Uninfected “mock” controls had an equal volume of cell culture media added. Viral adsorption was left for 16 hours, then the inoculum was removed, the cells were washed, and fresh media added. Infections were left for a further 3 days before samples were taken. Four lyssavirus strains were utilized in this study: CVS-11, a laboratory adapted strain, and 3 field isolates (Sundaramoorthy et al., 2020a). The field isolates included Australian bat lyssavirus isolated from a horse (H.ABLV), and rabies viruses isolated from a Canadian silver-haired bat (SHBRV) and a Zimbabwean dog (Z.Dog). Three separate differentiation and infection experiments were conducted per lyssavirus strain, each with three technical replicates. Sequencing analyses were performed from 1-3 technical replicates in each of the three biological replicates.

### Immunoblotting

Immunoblotting was carried out using the BOLT pre-cast electrophoresis system (Invitrogen), where 20 µl of protein lysate was separated on 4-12% pre-cast NuPAGE Bis-Tris Midi Gels (Life Technologies) and transferred to PVDF (in full) membrane (ThermoFisher Scientific). Immunoblots were blocked with 3% w/v BSA in DPBS and incubated with primary antibodies for 1 hr, before detection with HRP-conjugated secondary antibodies (1:5000; BIO-RAD), and an ECL detection kit (Pierce). Primary antibodies: rabbit anti-rabies nucleoprotein (1:3000, in-house (Rahmadane et al., 2017); chicken anti-MAP2 (1:1000, ABCAM, cat#ab4674); rabbit anti-GFAP (1:1000, DAKO cat#Z0334); mouse anti-tubulin (1:500, Sigma cat#T8535).

### Exosome Isolation and RNA Extraction

Tissue culture supernatant was carefully removed from the neural cell cultures, then centrifuged at 300 x g for 10 min to pellet any cells or cell debris. Exosomes were isolated from 1 ml cell-free culture supernatant using Total Exosome Isolation Reagent (Invitrogen) as per the manufacturer’s protocol. Briefly, 500 µl of exosome isolation reagent was thoroughly mixed with 1 ml of cell-free supernatant and then incubated for 16 hr at 4°C. The reagent-supernatant mix was then centrifuged at 10,000 x g for 1 hr to pellet the exosomes. This method results the concentration of exosomes, however a small amount of other non-exosomal contaminants and protein aggregates may also be present (Zeringer et al., 2013; Li et al., 2017), the latter of which is removed during the RNA isolation process. The supernatant was discarded and exosomal RNA immediately extracted by adding the lysis buffer directly to the exosome pellet.

### RNA Extraction and Sequencing

Total RNA from human stem cell-derived neural cells and exosomes was isolated using the miRCURY RNA Isolation Kit-Cell and Plant (Exiqon, Copenhagen, Denmark) following the manufacturer’s protocol. RNA was quantitated using a spectrophotometer. cDNA libraries were constructed using the QIAseq miRNA Library Kit and QIAseq miRNA NGS 48 Index IL (Qiagen) as per the manufacturer’s protocol. For cell samples, 200 ng of total input RNA was used as the template with 16 cycles of library amplification. For exosome samples, 5 µl of eluted total RNA was used as the template with 22 cycles of library amplification. All libraries went through pre-sequencing quality control using the High Sensitivity DNA kit on the Bioanalyzer 2100 (Agilent) to ensure appropriate amplicon insert and minimal adapter carryover. Libraries were analyzed at the Australian Genome Research Facility (AGRF) for 100 bp single-end sequencing using the HiSeq 2500 (Illumina).

### Data Pre-Processing, Differential Expression, and Machine Learning Analysis

Pre-processing and downstream analysis was conducted as previously described (Farr et al., 2021). Briefly, adapters were trimmed [cutadapt (Martin, 2011)], reads underwent QC (FastQC, Babraham Bioinformatics), then were mapped and quantified [miRDeep2 (Friedlander et al., 2012)] against the miRBase human reference (version 22) (Kozomara et al., 2019). Read normalization and differential expression analysis was completed using DESeq2 (Love et al., 2014). FDR adjusted p-values < 0.05 were considered significant. Machine learning (ML) analysis was conducted using the scikit-learn (Pedregosa et al., 2011) module in Python. Highly correlated miRNAs (Pearson R of >0.8 or <-0.8) were removed, then the data was scaled using a robust scaling method (the median was removed, and the data scaled according to the interquartile range). Feature (miRNA) selection was performed using recursive feature elimination (RFE) based on a logistic regression classification model. The model was optimized then assessed by splitting the data up randomly into 70% labelled training data and 30% unlabelled test data, and the predicted classes of the test data samples were compared to the true classes. This process was repeated 1,000 times to ensure confidence in the classification performance. The logistic regression models were assessed for their accuracy (how many of the predictions were correct), precision (how many of the predicted positives were true positives), recall (how many of the true positives were found by the model), and receiver operating characteristic area under the curve (ROC AUC), which is a succinct metric to describe a binary classification model (Tribolet et al., 2020). All ML analysis was conducted by comparing uninfected mock samples with lyssavirus infected samples (all strains grouped together).

### RT-qPCR

Lyssavirus N protein RNA and 18S rRNA was quantified as previously described (Sundaramoorthy et al., 2020a). Briefly, 30 ng of total input RNA was converted to cDNA and amplified using the AgPath-ID One-Step RT-PCR kit (Applied Biosystems) as per the manufacturer’s instructions. RABVD1 primers and FAM probe (Nadin-Davis et al., 2009) were utilized to quantify CVS-11, SHBRV and Z.Dog strains, and the insectivorous ABLV primers and FAM probe (Foord et al., 2006) were utilized to quantify H.ABLV (both primer and probe sets were synthesized by Integrated DNA Technologies). Eukaryotic 18S rRNA Endogenous Control (VIC™/MGB probe, primer limited) primer/probe mix (Applied Biosystems) was used to measure 18S rRNA. Quantitative PCR was completed using the QuantStudio™ 6 Flex Real-Time PCR instrument (Applied Biosystems). Cycling conditions were as follows: 50°C for 2 min, 95°C for 10 min, followed by 40 cycles of 95°C for 15 s and 60°C for 1 min. The threshold for all reactions was set to 0.1. Lyssavirus nucleoprotein RNA levels were normalized to 18S rRNA. Undetectable results were reported with Ct of 40.

### Statistical Analysis

Statistical analyses were completed using the SciPy v1.6.0 (Virtanen et al., 2020) and scikit-posthocs v0.6.7 (Terpilowski, 2019) packages. All measurements were taken from distinct samples. Differences in lyssavirus N protein RNA expression were compared using Kruskal-Wallis H test with post-hoc Dunn’s multiple comparison test.

## Results

### MicroRNA Expression in a Neural Lyssavirus Infection Model

**Figure 1:**
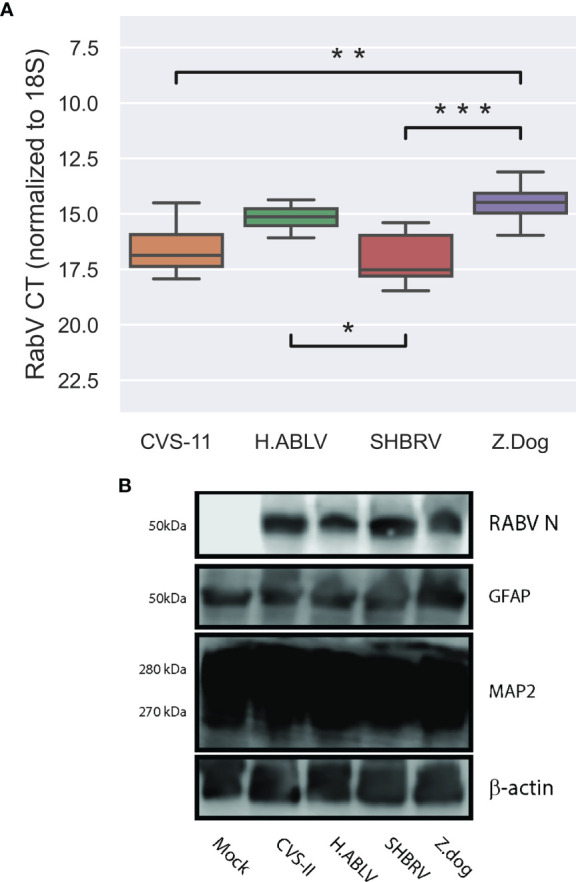
Lyssavirus infection of human stem cell derived neural cultures. **(A)** Boxplot of lyssavirus N protein RNA expression in CVS-11 (orange, n = 11), H.ABLV (green, n = 12), SHBRV (red, n = 10), and Z.Dog (purple, n = 12) infected HDF51i-509 neural cell cultures following 25-32 days *in vitro* differentiation. All sample numbers are technical replicates obtained from three separate biological experiments. Boxes are the 25th - 75th percentile, line is the median, and whiskers are 1.5x IQR. * p-value < 0.05, ** p-value < 0.01, *** p-value < 0.001. **(B)** Immunoblot of lyssavirus infection (RABV N protein) in HDF51i-509 stem cell-derived neural cultures expressing glial (GFAP), and neuronal (MAP2) markers.

In line with previous observations (Sundaramoorthy et al., 2020a), this neural culture displayed higher levels of neuronal marker MAP2, compared with glial marker GFAP, and can be infected all lyssavirus strains, with high levels of viral RNA and protein detectable 72 hours post-infection (h.p.i, **Figures 1A, B**). Despite an identical MOI for all infections, we observed significant differences in the detectable viral gene expression at 72 h.p.i (**Figure 1**), suggesting that some lyssavirus strains may replicate more efficiently than others in this cell model.

**Figure 2:**
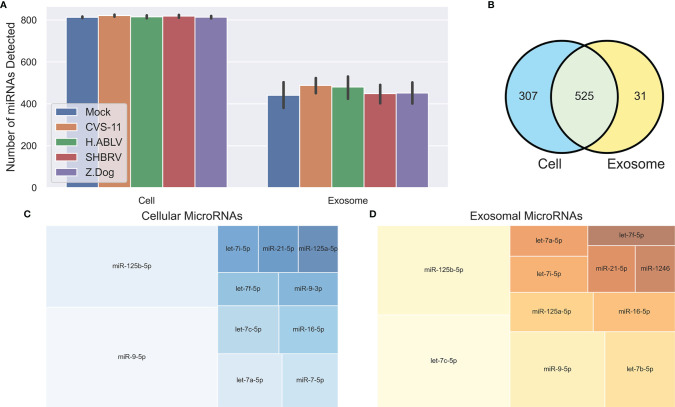
Overview of neuronal cellular and exosomal miRNAs. **(A)** Barplot of the number of mature miRNAs detected in cells or exosomes from each condition – Mock infection (blue, n = 6 cell, 9 exosome), CVS-11 (orange, n = 8 cell, 9 exosome), H.ABLV (green, n = 5 cell, 9 exosome), SHBRV (red, n = 5 cell, 9 exosome), and Z.Dog (purple, n = 6 cell, 9 exosome). All sample numbers are technical replicates obtained from three separate biological experiments. Error bars are 95% CI. **(B)** Venn diagram of the mature miRNAs detected in the neural cultures, exosomes or both. **(C, D)** Treemap plots displaying the most abundant miRNAs (each miRNA has > 2% of the total reads) in the neural **(C)** cells and **(D)** exosomes.

Small RNA NGS resulted in 13-19 million (average 16 million) raw reads for the cellular samples, and 15-33 million (average 18 million) raw reads for their corresponding exosome samples (submitted to the NCBI short read archive, BioProject PRJNA765814). Adapters were removed from the raw reads, and then the reads were subject to filtering based on length and quality, leaving 7-13 million cellular reads (average 9 million), and 3-14 million exosomal reads (average 8 million) for further analysis. A total of 832 mature cellular and 556 mature exosomal miRNAs were identified using miRDeep2 (**Figure 2A**), and their normalized reads were utilized for downstream analysis. Despite significant differences in viral gene expression, no cellular or exosomal miRNA displayed meaningful correlation with changes in viral RNA (Pearson R^2^ > 0.5), indicating that the degree of viral replication does not have a direct relationship with host miRNA expression. Predictably, there was considerable overlap between the miRNAs detected in the cellular and exosomal fractions, however 31 miRNAs were only found in secreted exosomes (**Figure 2B**). The most abundant cellular miRNA was miR-9-5p, followed by miR-125b-5p and let-7a-5p (**Figure 2C**). The most abundant exosomal miRNA was let-7c-5p, followed by miR-125b-5p and miR-9-5p (**Figure 2D**). Again, we saw overlap of highly expressed miRNAs (each with >2% of the total reads) between cell and exosome samples, except for two miRNAs in cellular samples (miR-7-5p and miR-9-3p), and two miRNAs in exosome samples (let-7b-5p and miR-1246).

### Lyssavirus Infection Induces Host miRNA Responses

**Figure 3:**
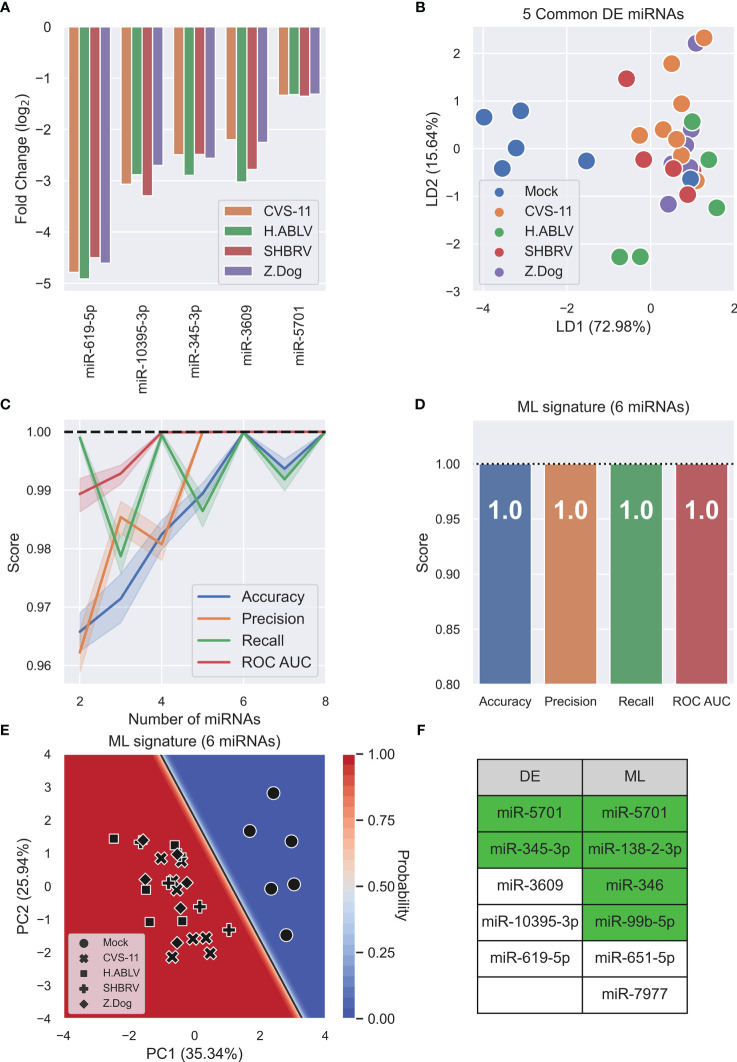
Lyssavirus infection results in cellular miRNA changes. **(A)** Fold change barplot of differentially expressed HDF51i-509 neural miRNAs (adjusted p-value < 0.05) common to CVS-11 (orange, n = 8), H.ABLV (green, n = 5), SHBRV (red, n = 5), and Z.Dog (purple, n = 6) compared with Mock infection (n = 6). All sample numbers are technical replicates obtained from three separate biological experiments. **(B)** Linear discriminant analysis (LDA) scatterplot using the 5 common DE miRNAs. Groups are Mock infection (blue), CVS-11 (orange), H.ABLV (green), SHBRV (red), and Z.Dog (purple). **(C)** Feature (miRNA) selection line plot showing the impact of increasing numbers of miRNAs on the performance of a logistic regression model. MicroRNAs were selected using recursive feature elimination to identify the most important miRNAs. Each combination of miRNAs was randomly assessed 1,000 times. Shaded areas are the 95% CI, and the dotted line is a perfect (100%) score. **(D)** Barplot showing the average performance metrics of the 6-miRNA ML signature in classifying mock infected and lyssavirus-infected neural cell culture. Error bars are the 95% CI after 1,000 random iterative assessments. Dotted line is 100%. **(E)** Decision boundary graph showing the logistic regression decision point (solid black line) and the probability neural cells are infected with lyssavirus (blue to red shading). Datapoints are mock infection (circles), CVS-11 (crosses), H.ABLV (squares), SHBRV (plusses), and Z.Dog (diamonds) neuronal cell samples. **(F)** Table listing miRNAs highlighted by differential expression (DE) and machine learning (ML) analysis. MicroRNAs highlighted in green have been previously associated with neuronal cell function, altered behavior, or both.

After DESeq2 normalization, a subset of 25 differentially expressed (DE) cellular miRNAs were found (false-discovery rate, FDR<0.05) after infection with at least one lyssavirus strain compared to uninfected mock controls (**Supplementary Table 1**). CVS-11 infection had a more widespread effect on miRNA expression than the field lyssavirus strains (21 differentially expressed miRNAs compared to 8-9 miRNAs in the field isolates), with 14 miRNAs changing only in response to this lab-adapted virus (**Supplementary Table 1**). Five miRNAs (miR-619-5p, -10395-3p, -345-3p, -3609, and -5701) decreased in response to all lyssavirus strains tested (**Figure 3A**). Supervised linear discriminant analysis (LDA) shows moderate separation between mock infection and lyssavirus samples using the 5 common DE miRNAs, however one mock sample clustered with lyssavirus samples (**Figure 3B**). Machine learning (ML) was then used as a multivariate analysis tool to identify miRNAs whose combined change in expression allowed lyssavirus infected cells to be distinguished from uninfected mock controls. ML identified 6 miRNAs (miR-5701, miR-138-2-3p, miR-346, miR-99b-5p, miR-651-5p, and miR-7977) (**Figure 3C**) that could distinguish lyssavirus infected neural cells with 100% accuracy, precision, recall, and ROC AUC (**Figure 3D**). Of these, only miR-5701 was also identified through DE analysis. These 6 ML miRNAs allow greater separation between the groups, and deliver a classification model with high confidence, as seen in the decision boundary graph (**Figure 3E**). Interestingly, ML analysis highlighted more miRNAs associated with neuronal cell function or altered behavior than DE analysis (4 and 2, respectively) (**Figure 3F**).

### Four Exosomal miRNAs Can Accurately Predict Neuronal Lyssavirus Infection

**Figure 4:**
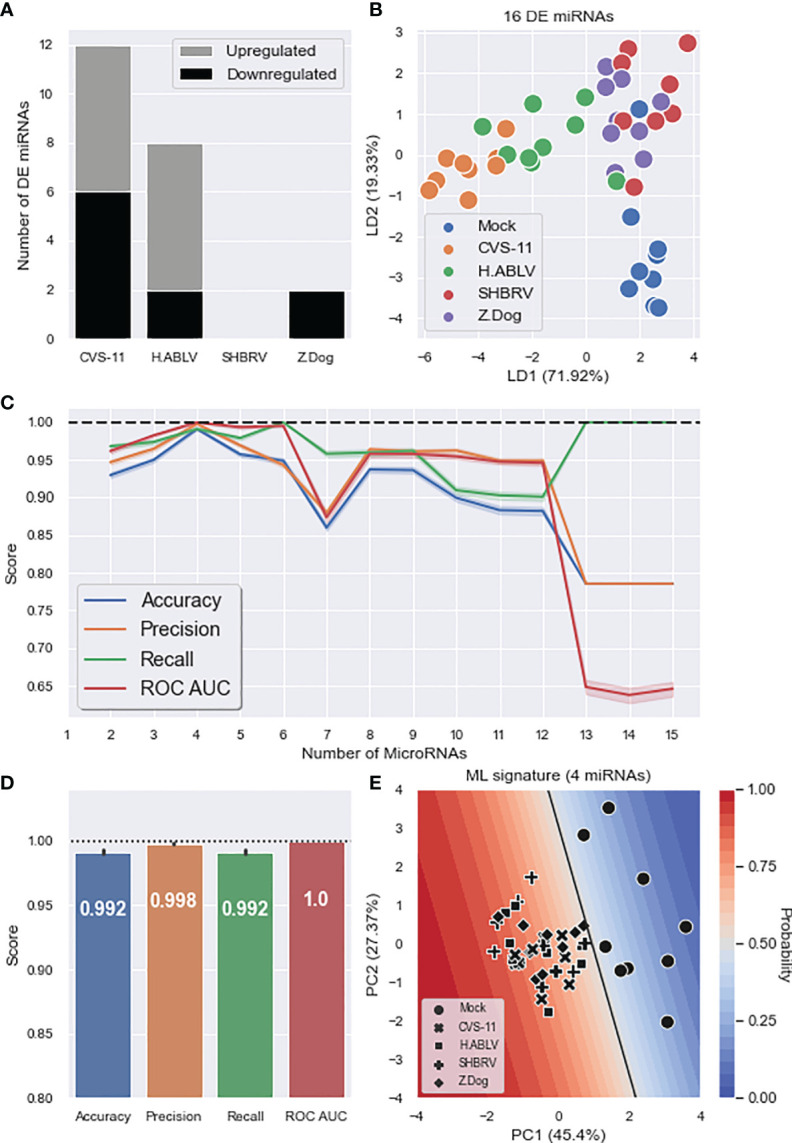
Exosomal miRNA composition changes during neuronal lyssavirus infection. **(A)** Stacked barplot showing the number of DE HDF51i-509 neural exosomal miRNAs (upregulated – grey, downregulated – black) identified in response to each lyssavirus strain compared to a mock infection. **(B)** Linear discriminant analysis (LDA) scatterplot using all 16 DE miRNAs identified in at least one lyssavirus strain. Groups are Mock infection (blue), CVS-11 (orange), H.ABLV (green), SHBRV (red), and Z.Dog (purple). N = 9 technical replicates in each group obtained from three separate biological experiments. **(C)** Feature (miRNA) selection lineplot showing the impact of increasing numbers of miRNAs on the performance of a logistic regression model. MicroRNAs were selected using recursive feature elimination to identify the most important miRNAs. Each combination of miRNAs was randomly assessed 1,000 times. Shaded areas are the 95% CI, and the dotted line is a perfect (100%) score. **(D)** Barplot showing the average performance metrics of the 6-miRNA ML signature in classifying mock infection and lyssavirus-infected neural cell culture. Error bars are the 95% CI after 1,000 random iterative assessments. Dotted line is 100%. **(E)** Decision boundary graph showing the logistic regression decision point (solid black line) and the probability neural cells are infected with lyssavirus based on the exosomal miRNA signature (blue to red shading). Datapoints are mock infection (circles), CVS-11 (crosses), H.ABLV (squares), SHBRV (plusses), and Z.Dog (diamonds) neural cell samples.

To investigate the use of cell-free miRNAs as biomarkers of lyssavirus infection, we profiled the changes in exosomal miRNAs released from this stem cell-derived neural model. Mirroring the cellular miRNA results, CVS-11 triggered the most diverse changes, with the abundance of 12 miRNAs (6 up, 6 down) significantly altered (**Figure 4A**). Surprisingly, SHBRV did not result in any DE miRNAs being detected, while only 2 DE miRNAs (both downregulated) were found in exosomes from Z.Dog infected neurons (**Figure 4A** and **Supplementary Table 2**). As there were no common DE miRNAs, the abundance of all 16 identified DE miRNAs was used in the supervised LDA analysis, which resulted in moderate separation between the uninfected mock and lyssavirus infected groups (**Figure 4B**). ML was then employed to identify a minimal exosomal miRNA signature that could be used to predict lyssavirus infection in the neural cells. The data was randomly split into discovery and validation sets, a logistic regression classification model was trained and tested, then this process was repeated 1,000 times to determine reproducibility. The most predictive miRNAs were selected using recursive feature elimination (**Figure 4C**). Four miRNAs (miR-25-3p, miR-26b-5p, miR-218-5p, miR-598-3p) were identified to be the optimal signature, resulting in 99.2% accuracy, 99.8% precision, 99.2% recall and 100% ROC AUC (**Figure 4D**). Interestingly, only miR-25-3p and miR-218-5p were identified using DE analysis; if using only these two DE miRNAs the accuracy of the ML model drops to 86.1%. Adding additional miRNAs into the signature did not improve performance, and more than 12 miRNAs resulted in the accuracy of the model falling significantly (**Figure 4C**). A decision boundary graph shows a clear separation between the uninfected mock samples and the lyssavirus groups (**Figure 4E**).

## Discussion

Evasion of the host immune response is a key feature of rabies pathogenesis, which has not only hindered the development of diagnostic tests for pre-clinical infection but has limited our understanding of the host-cell response at a cellular and molecular level. In concert, a lack of appropriate *ex-vivo* models for studying rabies infection in human neurons has contributed to the critical knowledge gap around the clinical pathogenesis of the virus. Here, we have utilized a previously described human stem cell-derived neural model (Sundaramoorthy et al., 2020a) to better explore host-cell and exosome associated miRNA expression during human rabies infection, and ascertain the use of miRNA expression as a reliable predictive biomarker of infection.

Several studies have previously examined lyssavirus-mediated miRNA changes in brain homogenates of infected mouse models (Han et al., 2011, Zhao et al., 2012a; Zhao et al., 2012b, Shi et al., 2014). However, despite recent advances in modelling neurotropic viruses in human neural stem cell systems (Tabari et al., 2020), the application of *in vitro* or human neural stem cell model systems has not been employed for improved differentiation between cellular or non-cellular (exosomal) miRNAs for lyssaviruses.

In this study, we also took the opportunity to compare differing bioinformatics approaches: conventional differential expression (DE) and machine learning (ML) analysis. DE is performed by comparing the expression levels of each miRNA between groups and using a statistical test to assess the probability of any difference happening by random chance. As there is usually a large number of comparisons (832 in the cell samples), stringent p-value corrections are often applied to control false positives. This approach effectively identifies large scale changes but can result in false negatives due to overly harsh p-value corrections. This potentially explains why no DE exosomal miRNAs were identified in human neural cultures infected with SHBRV (**Figure 4A**). DE also only looks at individual miRNA changes, with no regard for the complex molecular interplay which occurs in biological systems.

ML, on the other hand, attempts to build a model that results in the best classification prediction (in this case testing mock infection or infected status of cell cultures), usually in terms of accuracy, precision, recall or ROC AUC. Although a ML model can be constructed using a single miRNA, it is more often built using multiple miRNAs, utilizing their combined expression pattern, rather than looking at them individually. This will better take into consideration the complexity of the larger system and enable accurate predictive analysis. Unfortunately, ML can be challenging to implement, and its performance is often dependent on the amount of data available (more samples generally result in a stronger model). ML is yet to be become a default methodology within the biological sciences, although it is likely to become more widespread. Indeed, some miRNA studies have already implemented this approach (Duy et al., 2016; Farr et al., 2021; Wong et al., 2021).

By applying a multivariate, machine learning (ML) data analysis methodology, we have identified unique cellular and exosomal miRNA signatures of lyssavirus pathology that predict infection with 100% and 99.2% accuracy respectively (**Figures 3D**, **4D**). These signatures result in more distinct separation of uninfected and lyssavirus infected samples (**Figures 3B**, **4B**) and identified more miRNAs involved in neuronal cell fate and behavioral traits (**Figure 3F**), when compared to miRNAs highlighted through DE analysis.

Understanding the roles of these newly identified miRNAs in the host-cell infection response holds the potential to significantly improve our knowledge of the pathology and pathogenesis of viral disease. Here, ML analysis has highlighted both cell-associated and exosomal miRNAs associated with virus infection in neurons, that were not all detected using DE analysis. MicroRNA-5701 (found in both DE and ML analysis) regulates neuronal cell death through mitochondrial-lysosomal cross talk (Prajapati et al., 2018), but taken alone provides little insight into the pathogenic mechanism of the virus. However, when we start to consider other miRNAs identified in the associated ML signature (miR-99b-5p, miR-346, miR-5701, miR-138-2-3p, miR-651-5p, and miR-7977), we can begin to hypothesize on the molecular mechanisms underpinning viral pathogenesis in the neurons.

For example, many of the cellular and exosomal miRNAs identified through ML analysis have been previously associated with neuronal cell death and neurodegenerative disorders. MicroRNA-99b-5p and miR-5701 (ML cell signature) have established roles in neuronal cell death (Ye et al., 2015; Prajapati et al., 2018), neuroprotection (Altintas et al., 2016) and neuroregeneration (Cao et al., 2017), while miR-25-3p and miR-218-5p (exosome ML signature) have been shown to have neuroprotective effects in models of stroke (Kuang et al., 2020), epilepsy (Li et al., 2020) and Parkinson’s disease (Ma et al., 2021).

Whilst lyssavirus does not induce apoptosis in infected neurons, it has been shown that neurons activate a selective and compartmentalized SARM-1-mediated degeneration of their axons and dendrites in response to infection with different field strains of lyssavirus (Sundaramoorthy et al., 2020b). This response may act as a putative neuroprotective mechanism against the transport of the pathogen through the peripheral and central nervous system. A similar axonal destruction mechanism, called Wallerian degeneration, has also been reported in Amyotrophic lateral sclerosis (ALS) and Parkinson’s disease (White et al., 2019). Deletion of Dicer (a key protein in miRNA biogenesis) results in progressive axonal degeneration in the peripheral nervous system of mice (Li et al., 2018), indicating a role for miRNAs in regulating this cellular response. More research is needed to confirm the role of ML miRNA candidates such as miR-99b-5p, miR-5701, miR-25-3p and miR-218-5p in regulating neuronal degeneration during lyssavirus infection, however they are compelling candidates in this pathogenesis pathway and warrant further investigation.

Another sub-set of miRNAs identified by ML correlate to behavioral regulation by the brain. For instance, miR-138-2 (ML cell signature) is associated with panic disorder and directly targets the gamma-aminobutyric acid receptor *GABRA6*, a gene implicated in the etiology of anxiety disorders (Muinos-Gimeno et al., 2011). Overexpression of this miRNA in C57BL/6J mice lead to impaired learning and memory, and increased anxiety (Boscher et al., 2020). MicroRNA-598-3p (exosome ML signature) was also shown to change during hearing loss-related cognitive impairment (Mun et al., 2021). Circulating miR-26b-5p was altered in Alzheimer’s disease and the behavioral variant of Frontotemporal dementia, a condition that leads to behavioral disinhibition, impulsivity, and deficits in complex thinking (Denk et al., 2018). Finally, although not significantly correlated with impulsive traits, miR-346 (exosome ML signature) is located within *Neuroregulin-3*, a gene that has been shown to regulate impulsivity in mice (Loos et al., 2014; Pietrzykowski and Spijker, 2014) and promotes synapse formation in excitatory neurons (Muller et al., 2018). This miRNA was also reduced in extracellular vesicles in the forebrain of mice with chronic temporal lobe epilepsy (Gitai et al., 2020). Together, this cluster of miRNAs generated from the ML algorithm may point to a role of miRNAs in the behavioral alterations, confusion, and anxiety that is often seen in clinical rabies infections. Again, this would warrant a much deeper analysis, but provides a basis for furthering our understanding of the molecular mechanisms underpinning clinical signs and symptoms of the disease.

Interestingly, linear discriminant analysis (LDA) of the 16 exosome miRNAs obtained from DE analysis was able to highlight strain-specific miRNA changes, especially when comparing the lab-adapted CVS-11 strain and the field isolates. CVS-11 infection affected the expression of more distinct miRNAs than the field isolates, both within neural cell cultures and the exosomes they secrete (**Supplementary Tables 1**, **2**). This has important implications for studies exclusively employing lab-adapted strains for rabies disease modelling as these strains may not offer a realistic insight into rabies pathogenesis of field isolates. Indeed, attenuated lyssaviruses have been shown to activate the host immune response, rather than evade it (Wang et al., 2005), and CVS-11 specific miRNA responses may play a role in this divergent host-pathogen interaction, although further work is warranted to confirm this relationship.

Finally, exosome miRNA signatures have the potential to be explored as biomarkers for novel diagnostic tests against pre-clinical rabies infection. As previously mentioned, Rabies has an unusually long asymptomatic incubation period with little to no early immune activation, which has led to a gap in effective ante-mortem diagnostic testing protocols. Prophylactic treatment is effective but costly, and sometimes inaccessible. A tool to stratify infected patients and justify the cost versus benefit of therapeutic intervention would enable more efficient implementation, particularly throughout the developing world.

Prior to this study, only one paper has investigated the release of miRNAs into circulation following rabies infection (Han et al., 2011). Seven serum miRNAs were found to be DE in the study by (Han et al., 2011), none of which were identified by our analysis. Several reasons may have contributed to this discrepancy, including the use of an *in vivo* murine model of infection as opposed to/compared to our *in vitro* human neural culture system; serum versus cell culture supernatant sampling of putative miRNAs; low sampling numbers from the animal model which displayed extensive heterogeneity in viral RNA and protein expression in infected groups; and the timing of sampling – 1 week versus 72 hours post-inoculation. On this last point it is interesting to note that (Han et al., 2011) do state that the expression of the seven miRNAs identified was specific to the infected mice, regardless of infection stage between 1-3 weeks. However, from this study it is not known if the miRNA expression profile would be different at a time point earlier than 1 week post-inoculation.

From our study, the four exosomal miRNAs (miR-25-3p, miR-26b-5p, miR-218-5p, miR-598-3p) that were identified could be used to predict lyssavirus infection in the neural cells with >99% accuracy. Whilst this is promising foundational data upon which to develop a novel circulating biomarker, further work is still needed to ensure these miRNAs are detectable *in vivo* at clinically relevant time points. It would also be prudent to more accurately determine the level of expression of miRNAs in different nerve populations, such as the sensory and motor neurons of the peripheral nervous system and the differing classes of neurotransmitter-responsive nerves in the central nervous system. Given the supporting role of glial cells (astrocytes, oligodendrocytes and Schwann cells) in nerve function, damage and repair responses, the contribution of glial miRNAs to viral pathogenesis and neural system damage also warrants a more detailed investigation. Finding these signatures in accessible body fluids is also an essential step toward the establishment of this miRNA infection signature as a biomarker for *in vitro* diagnostics.

None-the-less, whilst optimization of novel biomarker signatures for viral diseases like rabies matures, new technologies are emerging in parallel that enable rapid, cost-effective exosome capture and miRNA detection (Tribolet et al., 2020; Mohammadi et al., 2021). Ideally, coupling these modern diagnostic platform technologies with a validated biomarker signature would eventually enable the use of miRNA biomarkers for rabies virus to become a reality.

In conclusion, this study has brought together protocols and experimental techniques across a range of specialisms (including stem cell biology, virology, bioinformatics and machine learning) to gain further insight into the neural response to rabies infection. We show that applying a machine learning approach to conventional bioinformatic assessment can improve the accuracy and detail of our datasets. In addition, information obtained from DE and ML analysis provides interesting insights into the molecular role and relationship of miRNA clusters during viral infection. It would be interesting, and pertinent, to examine these miRNA changes in other neurotropic infections to assess their specificity to rabies and their role in wider diseases states. Lastly, we have identified a novel exosomal miRNA signature can independently predict lyssavirus infected cells with >99% accuracy and provides a foundation for utilizing exosome miRNAs in the development of next-generation molecular diagnostics for rabies. Although still in the early discovery stage as an “ideal” biomarker for rabies, further research of this signature, or the methodologies used to find it, may eventually become the foundation upon which diagnostics, improved pre-clinical management and potentially new treatments are identified for the disease.

## Data Availability Statement

The original contributions presented in this study are publicly available at the NCBI Sequence Read Archive using BioProject PRJNA765814 (https://www.ncbi.nlm.nih.gov/bioproject/PRJNA765814).

## Author Contributions

RF, NG, VS, and DG performed the experiments. RF and CC analyzed the data. RF wrote the first draft. MD led and oversaw the project. MD, CC, CS, CO’B, and JB contributed to the conception and design of the study. All authors contributed to manuscript revision, read, and approved the submitted version.

## Funding

This study was supported through the Commonwealth Scientific and Industrial Research Organisation (CSIRO) Probing Biosystems Future Science Platform.

## Conflict of Interest

The authors declare that the research was conducted in the absence of any commercial or financial relationships that could be construed as a potential conflict of interest.

## Publisher’s Note

All claims expressed in this article are solely those of the authors and do not necessarily represent those of their affiliated organizations, or those of the publisher, the editors and the reviewers. Any product that may be evaluated in this article, or claim that may be made by its manufacturer, is not guaranteed or endorsed by the publisher.
